# Management and Outcomes of Intracranial Hemorrhage in Atrial Fibrillation Patients: Highlighting Practices in Saudi Arabia

**DOI:** 10.7759/cureus.82813

**Published:** 2025-04-22

**Authors:** Abdulaziz M Alghamdi, Osama A Alkulli, Feras Fatani, Fahad S Subahi, Abdullah F Tayeb, Arwa Alghamdi, Moajeb Alzahrani, Fahad Almehmadi

**Affiliations:** 1 College of Medicine, King Saud Bin Abdulaziz University for Health Sciences, Jeddah, SAU; 2 Research Office, King Abdullah International Medical Research Center, Jeddah, SAU; 3 Department of Neurosurgery, King Abdulaziz Medical City, Ministry of National Guard Health Affairs, Jeddah, SAU; 4 Department of Cardiac Sciences, King Faisal Cardiac Center, King Abdulaziz Medical City, Ministry of National Guard Health Affairs, Jeddah, SAU

**Keywords:** anti-coagulant, anti-platelet, atrial fibrillation, blood thinners, intracranial hemorrhage, medication holiday

## Abstract

Objectives: Atrial fibrillation (Afib) requires anticoagulation to prevent strokes; however, it concurrently increases the risk of bleeding, including intracranial hemorrhage (ICH). Balancing thromboembolism prevention with bleeding risk is challenging, and guideline variations add uncertainty. Evaluating patient factors and ICH management is key to optimizing treatment and outcomes.

Methods: This is a retrospective cohort study conducted in King Abdulaziz Medical City in Jeddah, Saudi Arabia. This design is particularly well-suited for studying rare events like ICH, as it enables the inclusion of a larger sample size over an extended time period without the need for a long follow-up. Patients were identified through medical records of those with Afib on anticoagulation who developed ICH, confirmed by brain CT. The primary endpoint was to evaluate the management, outcome, and prognosis of ICH in these patients. The secondary endpoint was to assess the association between clinicopathological features and in-hospital mortality.

Results: A total of 36 patients were included in this study. Patients who were ≥ 70 years old accounted for 52.7%, and males constituted 61.1% of the patients. Spontaneous ICH was seen in 72.2%, while the rest were traumatic in origin. Conservative management was done in 80.5%; 69.4% had their Afib medication ceased upon admission, and only 66.6% of those had their Afib medications resumed. The factors associated with mortality during hospital admission included higher BMI (30.2 (26.3-33.1) vs. 25.1 (22.1-29.2), P = 0.0255), diabetes (14 (82.3%) vs. 8 (42.1%), P = 0.0134), higher International Normalized Ratio (INR) (1.8 (1.2-2) vs. 1.2 (1.1-1.3), P = 0.0356), spontaneous ICH (15 (88.2%) vs. 11 (57.8%), P = 0.0425), and Glasgow Coma Scale (GCS) ≤ 8 (15 (88.2%) vs. 4 (21.0%), P = 0.0002). Regarding the outcome, 47.2% passed away during their hospital stay. Upon discharge, 78.9% had a GCS score of ≥ 14; apixaban was the most common medication prescribed (42.1%). The follow-up periods of the discharged patients had a median of 445 days; 33.3% passed away, while only 5.5% of them developed a recurrent ischemic stroke.

Conclusion: Our findings revealed that ICH in Afib patients is associated with high mortality and overall poor prognosis. There is a clear need for standardized management guidelines. Further studies are essential to establish evidence-based recommendations and reach reliable conclusions to improve patient outcomes.

## Introduction

Atrial fibrillation (Afib) is a heart condition characterized by an irregular and abnormally high heart rate, usually higher than 100 beats per minute. It is the most common form of sustained cardiac arrhythmia in clinical practice, with an increased risk of death, heart failure, hospitalization, and thromboembolic events [1-3]. In 2010, according to Chugh et al., the global prevalence of Afib was 20.9 million for males and 12.6 million for females [4]. During Afib, the atria beat chaotically and irregularly out of sync with the ventricles, which causes the ventricles not to fill or pump enough blood to the lungs and body, subsequently leading to heart-failure-like symptoms and blood pooling in the atria, which increases the risk of clot formation and ischemic stroke. Afib can be valvular (VAfib), when a heart valve condition causes arrhythmia, or non-valvular (NVAfib), when caused by other systemic factors such as hypertension or coronary artery disease [5]. The management of NVAfib generally focuses on symptom relief and reducing the risk of thromboembolic events, often through anticoagulation with direct oral anticoagulants (DOACs). In contrast, VAfib, particularly in patients with mechanical heart valves, typically requires the use of warfarin due to its more established efficacy in preventing thromboembolism in this subgroup. According to the American College of Cardiology (ACC), long-term antithrombotics are recommended in high-risk populations with a CHA_2_DS_2_-VASc score of ≥2 in men and ≥3 in women [6].

Long-term antithrombotic use is associated with a high risk of major hemorrhagic complications [7]. Intracranial hemorrhage (ICH) has been documented in the literature as a possible complication in Afib patients taking antithrombotics, with an incidence of 0.8% per year, with most cases being spontaneous and intraparenchymal [8]. Moreover, >50% of ICH among Afib patients is fatal [9]. Generally, some antithrombotics are considered safer than others for patients with Afib. For example, a study found that using apixaban is preferable over warfarin as it helps prevent stroke and causes fewer bleeding complications. However, the chance of developing ICH is still high [10].

However, another study found that the use of apixaban or warfarin did not change the risk of major bleeding or death. The same study revealed that the risk of death due to ICH is higher than that due to stroke or myocardial infarction [11]. Usually, the initial step in managing ICH in patients taking antithrombotics is to discontinue antithrombotic therapy immediately to prevent hematoma expansion [12]. However, in Afib patients, stopping the medication increases the risk of developing emboli and ischemic stroke [6]. According to a questionnaire-based study, most neuro-specialists tended to stop warfarin at first, normalize the International Normalized Ratio (INR) upon admission, and then restart the medications again; however, the strategies to normalize INR and restart warfarin therapy varied greatly and depended on each physician's decision [13]. Furthermore, the use of different types of antithrombotics among ICH survivors can lead to different outcomes. A systematic review revealed that restarting vitamin K antagonists resulted in better outcomes than antiplatelet or non-reusing anticoagulants for ischemic stroke. At the same time, no significant changes were observed in the ICH recurrence [14].

As it is challenging to manage ICH in Afib patients due to opposing factors, since the discontinuation of antithrombotic therapy results in more ischemic strokes, physicians, in some situations, are limited to doing so as the continuation of antithrombotic therapy can further worsen ICH. The clinical implications of Afib's pathophysiology concerning ICH management are particularly significant. In Afib, irregular atrial activity and impaired blood flow in the heart increase the formation of thrombi, which poses a heightened risk of stroke. However, this need for thromboprophylaxis directly conflicts with the management of ICH, where anticoagulation therapy needs to be halted to prevent further bleeding. This balancing act between preventing ischemic stroke through anticoagulation while managing the risk of exacerbating ICH with continued antithrombotic use poses significant clinical challenges. Moreover, the variability in physician approaches to managing such cases further complicates optimal treatment. In Saudi Arabia, there are no clear guidelines that physicians can follow in such cases, which can further complicate their management. The lack of formal guidelines likely leads to inconsistent approaches in clinical practice, contributing to potential variations in patient outcomes and the quality of care. In this study, we aimed to evaluate the management, outcomes, and prognosis of ICH in patients with Afib.

## Materials and methods

This retrospective study was conducted at King Abdulaziz Medical City in Jeddah, Saudi Arabia, to evaluate the management, outcomes, and prognosis of ICH in patients with Afib. The secondary objective was to assess the factors associated with mortality during hospital admission. This study design was selected because it is the most feasible and cost-effective option, as prospective studies would require many years of follow-up due to the rarity of the disease.

All patients aged 18 years or older diagnosed with Afib who experienced at least one episode of ICH between June 2016 and June 2023 were included in this study. This time frame was chosen because, prior to June 2016, the institution utilized paper-based records, which were less reliable and had lower-quality documentation. Starting in June 2016, the institution transitioned to a computer-based documentation system, enhancing the accuracy and reliability of patient records. Patients with a history of ICH before the diagnosis of Afib and those with incomplete or insufficient data were excluded. The participants' data were extracted and analyzed retrospectively from the hospital's electronic patient files. Ethical approval was obtained from King Abdullah International Medical Research Center in Jeddah, Saudi Arabia, under protocol number NRJ23J/245/09.

The data collection sheet comprised four sections: demographic data, ICH presentation, patients' hospital course, and outcomes and prognosis. The first section aimed to gather demographic data and background information on Afib, including variables such as sex, age, height, weight, comorbidities, Afib type, medications, HAS-BLED (hypertension, abnormal renal/liver function, stroke, bleeding) score, and CHADS_2_ score. The second section assessed ICH presentation details, including creatinine clearance, INR levels, etiology of ICH, symptoms, Glasgow Coma Scale (GCS) score, type of ICH, maximal diameter of the ICH, and, if applicable, maximal diameter of the residual ICH postoperatively. The maximal diameter of the hematoma was measured utilizing CT imaging. The third section details the patient's hospital course, including admission and discharge dates, length of hospital stay, discontinuation and resumption of anticoagulants, complications, ICH recurrence, and patient characteristics at discharge. The final section of the data collection sheet evaluated outcomes and prognosis during at least one year of follow-up after discharge, including the follow-up period, complications, recurrence, and mortality, if applicable.

The data were entered into an Excel spreadsheet (Microsoft Corporation, Redmond, Washington) and analyzed exclusively by the research team. The analysis was performed using John's Macintosh Project (JMP) Pro software version 15 (IMP Statistical Discovery LLC, Cary, North Carolina). Frequencies and percentages were used to present categorical variables, whereas medians and interquartile ranges were used for continuous variables. The association between two categorical variables was assessed using the chi-square test. For the association between numerical and categorical variables, we employed a t-test. Statistical significance was set at P < 0.05, which was considered statistically significant, and the corresponding 95% confidence interval (CI) was set.

## Results

Demographics and baseline characteristics

A total of 36 patients fit our criteria and were included in this study. Males constituted 61.1% of the patients, and 52.7% of the patients were aged ≥ 70 years. Hypertension accounted for 83.3% of the patients. A previous history of ischemic stroke or transient ischemic attack was present in 30.5% of the patients, and a previous history of hemorrhagic stroke or internal bleeding was present in only 5.5% of the patients. Regarding the type of Afib, 63.8% of patients had NVAfib. Warfarin was the most used Afib medication, with a percentage of 50.0%, followed by apixaban, with a percentage of 33.3%. The HAS-BLED and CHADS_2_ scores had medians of 3 (2-5) and 4 (3-6), respectively. The remaining demographic and baseline characteristics are shown in Table 1.

**Table 1:** Demographics and baseline characteristics of the patients. n: Number, BMI: Body Mass Index, IQR: Interquartile Range, TIA: Transient Ischemic Stroke, Afib: Atrial Fibrillation

Variables	Results (n=36)
Gender, n (%)	
Males	22 (61.1)
Females	14 (38.8)
Age, n (%)	
<70	17 (47.2)
≥70	19 (52.7)
BMI, median (IQR)	27.6 (23.6-32.3)
Comorbidities, n (%)	
Hypertension	30 (83.3)
Diabetes	22 (61.1)
Coronary artery disease/heart failure	22 (61.1)
Renal dysfunction	13 (36.1)
Liver disease	5 (13.8)
Previous ischemic stroke/TIA, n (%)	11 (30.5)
Previous hemorrhagic stroke/internal bleeding, n (%)	2 (5.5)
Smoker, n (%)	3 (8.3)
Type of Afib, n (%)	
Non-valvular	23 (63.8)
Valvular	13 (36.1)
Used medications, n (%)	
Warfarin	18 (50.0)
Apixaban	12 (33.3)
Aspirin	2 (5.5)
Plavix	2 (5.5)
Enoxaparin	2 (5.5)
Diltiazem	2 (5.5)
Amiodarone	2 (5.5)
Heparin	1 (2.7)
Digoxin	1 (2.7)
None	4 (11.1)
HAS-BLED score, median (IQR)	3 (2-5)
CHADS_2_ score, median (IQR)	4 (3-6)

ICH presentations

Upon presenting to the hospital with ICH, a decreased level of consciousness was the most common presenting symptom (77.7%), followed by headache (36.1%). Spontaneous ICH was observed in 72.2% of patients, while the rest were traumatic in origin. Approximately half of the patients (52.7%) had a GCS score of ≤8 upon presentation. The patients had a median of 70.5 mL/minute (38-88.7) creatinine clearance and a median of 1.2 (1.1-1.8) INR. Intraparenchymal ICH had the highest percentage (33.3%) among the cases, followed by intraparenchymal ICH with intraventricular extension (19.4%). Most ICH cases were acute in age, accounting for 75% of all cases. The median maximal diameter size of the ICH was 27 mm (14.2-38.2). The remaining ICH presentation data are presented in Table 2.

**Table 2:** Presentation and intracranial hemorrhage details. n: Number, IQR: Interquartile Range, ICH: Intracranial Hemorrhage, GCS: Glasgow Coma Scale

Variables	Results (n=36)
Etiology of ICH, n (%)	
Spontaneous	26 (72.2)
Traumatic	10 (27.7)
Type of trauma, n (%)	
Fall	7 (70.0)
Motor vehicle accident	3 (30.0)
Presenting symptoms, n (%)	
Decreased level of consciousness	28 (77.7)
Headache	13 (36.1)
Focal neurological symptoms	12 (33.3)
Nausea/vomiting	4 (11.1)
Dizziness	4 (11.1)
GCS on presentation, n (%)	
≥14	11 (30.5)
9-13	6 (16.6)
≤8	19 (52.7)
Creatinine clearance, median (IQR)	70.5 (38-88.7)
INR, median (IQR)	1.2 (1.1-1.8)
Type of ICH, n (%)	
Intraparenchymal	12 (33.3)
Intraparenchymal with intraventricular extension	7 (19.4)
Subdural	6 (16.6)
Intraventricular	5 (13.8)
Subarachnoid	3 (8.3)
Hemorrhagic transformation of an ischemic stroke	2 (5.5)
Epidural	1 (2.7)
Age of ICH, n (%)	
Acute	27 (75.0)
Subacute	6 (16.6)
Acute on top of chronic	3 (8.3)
The maximal diameter size of the ICH, median (IQR) in mm	27 (14.2-38.2)

Management, outcomes, and prognosis

Most patients (69.4%) had their Afib medication discontinued upon admission to the hospital. Only 66.6% of those who had their Afib medication eventually resumed. The median length of medication holidays was 14 (7.5-38) days. Conservative management was performed in 80.5% of cases; the rest underwent surgical management for ICH. Almost half of the patients (47.2%) died during their hospital stay, with a median time from admission to mortality of 26 (9.5-77.5) days. Approximately half of the patients (52.7%) were eventually discharged from the hospital, and 78.9% had a GCS score of ≥14. Apixaban was the most common medication prescribed to patients upon discharge (42.1%). The median length of hospital stay for all patients was 28 (7.2-80) days. The remainder of the hospital course data management is shown in Table 3.

**Table 3:** Hospital stay details. n: Number, IQR: Interquartile Range, ICH: Intracranial Hemorrhage, GCS: Glasgow Coma Scale, Afib: Atrial Fibrillation

Variables	Results (n=36)
Discontinuation of Afib medication upon admission, n (%)	25 (69.4)
Resumption of Afib medication after being discontinued, n (%) (n= 25)	17 (68.0)
Length of medication holiday, median (IQR) in days (n= 25)	14 (7.5-38)
*Management, n (%)*	
Conservative	29 (80.5)
Decompressive Hemicraniectomy	4 (11.1)
External Ventricular Device	3 (8.3)
*Complication during admission, n (%)*	
Mortality	17 (47.2)
Recurrent ICH	0
Recurrent Ischemic stroke	0
None	19 (52.7)
Time from admission to mortality, median (IQR) in days (n= 17)	26 (9.5-77.5)
*GCS on discharge, n (%) (n= 19)*	
≥14	15 (78.9)
9-13	3 (15.7)
≤8	1 (5.2)
*Medication on discharge, n (%) (n= 19)*	
Apixaban	8 (42.1)
Enoxaparin	2 (10.5)
Warfarin	2 (10.5)
Aspirin	1 (5.2)
Plavix	1 (5.2)
Digoxin	1 (5.2)
Diltiazem	1 (5.2)
Clopidogrel	1 (5.2)
None	4 (21.0)
Length of hospital stay, median (IQR) in days	28 (7.2-80)

Bivariate analysis of the factors associated with mortality during hospital admission revealed that higher BMI (30.2 (26.3-33.1) vs. 25.1 (22.1-29.2), P =.0255), diabetes (14 (82.3) vs. eight (42.1), P = .0134), higher INR upon presentation (1.8 (1.2-2) vs. 1.2 (1.1-1.3), P = .0356), spontaneous ICH (15 (88.2) vs. 11 (57.8), P = .0425), and GCS scores of ≤8 upon presentation (15 (88.2) vs. four (21.0), P = .0002) were significantly associated with mortality during hospital admission. Other factors, such as discontinuation of Afib medication, did not show any statistical significance (Table 4).

**Table 4:** Comparison of the associated factors with mortality during admission. * The chi-square test was used to evaluate the association between the different variables and mortality during admission. A P-value of <0.05 was considered statistically significant. n: Number, IQR: Interquartile Range, ICH: Intracranial Hemorrhage, GCS: Glasgow Coma Scale, Afib: Atrial Fibrillation, INR: International Normalized Ratio, TIA: Transient Ischemic Stroke

Variables	Made it outside the hospital (n=19)	Mortality during admission (n=17)	P-value*
Gender, n (%)			
Males	13 (68.4)	9 (52.9)	0.341
Females	6 (31.5)	8 (47.0)	
Age, n (%)			
<70	9 (47.3)	8 (47.0)	0.985
≥70	10 (52.6)	9 (52.9)	
BMI, median (IQR)	25.1 (22.1-29.2)	30.2 (26.3-33.1)	0.0255
Comorbidities, n (%)			
Hypertension	16 (84.2)	14 (82.3)	0.881
Diabetes	8 (42.1)	14 (82.3)	0.013
Coronary artery disease/heart failure	11 (57.8)	11 (64.7)	0.675
Renal dysfunction	8 (42.1)	5 (29.4)	0.428
Liver disease	3 (15.7)	2 (11.7)	0.727
Type of Afib, n (%)			
Non-valvular	12 (63.1)	11 (64.7)	0.923
Valvular	7 (36.8)	6 (35.2)	
HAS-BLED score, median (IQR)	4 (3-5)	3 (2-5.5)	0.371
CHADS_2_ score, median (IQR)	4 (3-6)	5 (3-6.5)	0.208
Previous ischemic stroke/TIA, n (%)	6 (31.5)	5 (29.4)	0.8879
Previous hemorrhagic stroke/internal bleeding, n (%)	2 (10.5)	0	0.168
Creatinine clearance, median (IQR)	82 (38-89)	66 (36-81.5)	0.401
INR, median (IQR)	1.2 (1.1-1.3)	1.8 (1.2-2)	0.035
Etiology of ICH, n (%)			
Spontaneous	11 (57.8)	15 (88.2)	0.042
Traumatic	8 (42.1)	2 (11.7)	
Maximum diameter of ICH, median (IQR)	24 (13-31)	35 (16.5-61.5)	0.051
GCS, n (%)			
≥14	10 (52.6)	1 (5.8)	>0.001
9-13	5 (26.3)	1 (5.8)	
≤8	4 (21.0)	15 (88.2)	
Discontinuation of Afib medication upon admission, n (%)	14 (74.6)	11 (64.7)	0.559

The median follow-up period of the 19 discharged patients was 445 (145.5-1252.2) days. During the follow-up period, 33.3% died, and 5.5% had recurrent ischemic stroke (Table 5). Detailed data on patient demographics, presentations, management, and hospital courses are shown in Table 6.

**Table 5:** Follow-up details. n: Number, IQR: Interquartile Range, ICH: Intracranial Hemorrhage

Variables	Results (n=19)
Follow-up period, median (IQR) in days	445 (145.5-1252.2)
Mortality during follow-up, n (%)	6 (33.3)
Recurrent ICH during follow-up, n (%)	0 (0.0)
Recurrent strokes during follow-up, n (%)	1 (5.5)

**Table 6:** Detailed data on patient demographics, presentations, management, and hospital courses. Afib: Atrial Fibrillation, IQR: International Normalized Ratio, ICH: Intracranial Hemorrhage, GCS: Glasgow Coma Scale, M: Male, F: Female, Nonvalv: Nonvalvular, Valv: Valvular, S: Spontaneous, T: Traumatic, C: Conservative, DHC: Decompressive Hemicraniectomy, EVD: External Ventricular Device

Variable/case number	Gender/age	Afib type	Afib medications	HAS-BLED/CHADS_2_	INR	ICH etiology	ICH type	GCS on presentation	Discontinuation of Afib medication upon presentation	Resumption of Afib medications	Length of medication holiday, in days	Management	Complications during hospital stay	Length of hospital stay, in days
1	F/80	Nonvalv	Apixaban, Aspirin, Diltiazem	4/4	1.2	T	Acute intraparenchymal with intraventricular extension	13	Yes	-	6	C	None	10
2	M/87	Nonvalv	Digoxin	2/3	1.1	T	Subacute subdural	3	No	-	-	C	None	50
3	M/66	Nonvalv	Warfarin	3/3	1.2	S	Acute intraparenchymal	13	Yes	Yes	124	C	None	30
4	M/73	Nonvalv	Warfarin	2/5	1.2	S	Acute intraventricular	3	No	-	-	C	Mortality	1110
5	M/74	Nonvalv	Amiodarone	2/3	1.8	T	Acute on top of chronic subdural	7	Yes	No	-	C	Mortality	197
6	F/59	Nonvalv	Apixaban	2/3	1.1	T	Acute subdural	15	Yes	Yes	9	C	None	7
7	M/76	Nonvalv	None	2/2	1.8	T	Acute subdural	4	No	-	-	DHC	None	198
8	M/93	Valv	Warfarin	7/7	1	T	Subacute intraventricular	12	Yes	Yes	19	C	None	39
9	F/79	Valv	Apixaban	7/4	1.2	T	Acute epidural	15	Yes	Yes	16	C	None	1
10	M/43	Valv	Warfarin	2/3	1.8	S	Acute intraparenchymal	15	Yes	Yes	13	C	Mortality	19
11	M/64	Valv	Apixaban	3/4	1	S	Acute intraparenchymal	14	Yes	No	-	C	None	3
12	M/50	Valv	Warfarin, Amiodarone	2/3	3.2	S	Acute intraparenchymal with intraventricular extension	6	Yes	Yes	9	EVD	Mortality	197
13	F/64	Valv	Heparin	3/5	1.2	S	Acute intraparenchymal	3	Yes	No	-	DHC	Mortality	8
14	F/74	Valv	Apixaban	5/7	1	S	Acute on top of chronic intraventricular	9	Yes	No	-	C	Mortality	112
15	M/50	Valv	Apixaban	3/2	1.4	T	Subacute subarachnoid	8	No	-	-	C	None	37
16	F/65	Valv	Warfarin, Plavix	6/6	1.1	T	Acute hemorrhagic transformation of an ischemic stroke	3	No	-	-	C	Mortality	32
17	M/61	Valv	Aspirin, Warfarin	5/6	1.3	S	Acute subdural	15	Yes	Yes	25	C	None	14
18	M/49	Nonvalv	Warfarin, Enoxaparin	2/2	1.2	S	Acute subarachnoid	7	Yes	Yes	6	C	None	111
19	F/69	Nonvalv	Warfarin, Enoxaparin	6/5	4.8	S	Acute subarachnoid	8	Yes	Yes	57	C	Mortality	107
20	M/69	Valv	Warfarin	6/4	2	S	Acute intraparenchymal	3	Yes	Yes	23	DHC	Mortality	41
21	M/72	Nonvalv	Warfarin	4/4	1.1	T	Subacute intraparenchymal	15	Yes	Yes	2	C	None	7
22	F/54	Valv	Warfarin	3/4	1.3	S	Acute intraparenchymal	15	Yes	Yes	5	C	None	26
23	M/78	Nonvalv	Apixaban	5/3	1.2	S	Acute intraparenchymal	15	Yes	Yes	89	C	None	23
24	F/69	Valv	Apixaban, Plavix	5/6	1.3	S	Acute hemorrhagic transformation of an ischemic stroke	12	No	-	-	C	None	14
25	M/78	Nonvalv	None	2/2	1.9	S	Acute intraventricular	7	No	-	-	C	Mortality	60
26	M/62	Nonvalv	None	3/5	1.2	S	Acute intraparenchymal with intraventricular extension	6	No	-	-	EVD	Mortality	151
27	F/74	Nonvalv	Warfarin	6/4	2	S	Acute intraparenchymal	4	Yes	No	-	C	Mortality	44
28	F/65	Nonvalv	Warfarin	3/7	1.2	S	Acute intraparenchymal with intraventricular extension	3	Yes	No	-	C	Mortality	12
29	F/81	Nonvalv	Warfarin	3/7	1	S	Acute intraparenchymal with intraventricular extension	4	No	-	-	EVD	Mortality	25
30	M/78	Nonvalv	Warfarin	5/5	3.2	S	Acute intraparenchymal	3	Yes	No	-	C	Mortality	1
31	F/84	Nonvalv	Warfarin	3/7	2	S	Acute intraparenchymal with intraventricular extension	3	Yes	No	-	C	Mortality	2
32	M/83	Nonvalv	Apixaban	3/6	1	S	Acute intraparenchymal with intraventricular extension	15	Yes	Yes	51	EVD	None	80
33	M/68	Nonvalv	Apixaban, Diltiazem	7/3	1.9	S	Subacute intraparenchymal	15	Yes	Yes	13	C	None	80
34	F/78	Nonvalv	Warfarin	6/9	1.1	S	Subacute intraparenchymal	15	No	-	-	C	None	3
35	M/80	Nonvalv	Apixaban	2/3	1.3	S	Acute intraventricular	4	No	-	-	C	Mortality	3
36	M/83	Nonvalv	Apixaban	4/4	1.3	S	Acute on top of chronic subdural	11	Yes	Yes	14	C	None	6

## Discussion

In this observational cohort study, we evaluated the management, outcomes, and prognosis of ICH in 36 patients with Afib. Most patients were managed conservatively and had their Afib medications discontinued upon admission. In addition, almost half of the patients died during hospitalization, while the other half were later discharged from the hospital. The decision to resume Afib medication was made in most of the discharged patients with no recurrent ICH in at least one year of follow-up. These findings suggest the presence of a notable variation in the management of ICH in Afib patients in the absence of relied-upon guidelines to follow. Variations in management can easily be evoked in this group of patients because they usually have a multidisciplinary team involved in their medical treatment.

Although the variations in ICH management in Afib patients in this study did not follow a specific pattern, some observations are worth mentioning. As mentioned earlier, upon admission to the hospital, most patients had their Afib medication stopped. Similar to our findings, Lopes et al. illustrated that vitamin K or other drugs were administered to individuals with acute ICH who had access to hospitals to halt bleeding [8]. Ghenbot et al. demonstrated the same pattern of management in 28 NVAfib patients with traumatic ICH [9,15,16]. However, the reasons for not discounting Afib medications in the remaining patients in our study remain unclear.

According to Grysiewicz et al., early treatment measures include anticoagulant withdrawal, reversal of coagulopathy, management of blood pressure and glycemia, intubation, external ventricular drainage or invasive monitoring, and treatment of intracranial pressure irrespective of the anticoagulant used [9]. Furthermore, most ICHs in our study were treated conservatively without surgical intervention. Similar to our findings, Lopes et al. reported that most patients were conservatively treated [8].

In our study, half of the patients survived and were eventually discharged from the hospital, indicating a high mortality rate. High BMI, diabetes, higher INR upon presentation, spontaneous ICH, and GCS scores ≤8 upon presentation were found to be significantly associated with mortality during hospital admission. Other studies also reported high mortality rates in their patients, showing a pattern of poor outcomes in patients with ICH [8,17].

The resumption of oral antithrombotic drugs in Afib patients after ICH development remains debatable. However, this study indicates that most individuals who discontinued their Afib medications resumed upon discharge. Analogous to this result, Lin et al. illustrated that patients who are at a high risk of thromboembolism and have ICH should be resumed on oral anticoagulants [18].

Apixaban was the prevailing medication prescribed to patients upon discharge. No recurrent ICH occurred during a follow-up period of at least one year. Similar to the current study are Lin et al., Cordonnier et al., and Steiner et al., which demonstrated that non-vitamin K antagonist oral anticoagulants are favored over warfarin among anticoagulants due to their ability to decrease significant bleeding events and enhance survival rates [18-22].

A randomized clinical trial involving 9081 patients with Afib who were prescribed warfarin and 9120 who were prescribed apixaban had a follow-up period of 1.8 years. Among those taking apixaban, 53 patients experienced ICH, and 23 died within the 30-day follow-up. In contrast, among the patients taking warfarin, 123 experienced ICH, with 52 deaths within the same follow-up period. However, there was no difference in the incidence of major bleeding events between the two anticoagulant medications. Additionally, major bleeding during anticoagulant use is associated with an increased risk of death [11].

Another observational cohort study illustrated the outcomes of warfarin resumption after traumatic ICH or hemorrhagic stroke in 2415 Afib patients. Among the patients with hemorrhagic stroke, resuming warfarin therapy was associated with a lower rate of ischemic stroke or systemic embolism and an increased rate of recurrent ICH than not resuming warfarin therapy. In patients with traumatic ICH, resuming warfarin therapy was also associated with a lower rate of ischemic stroke or systemic embolism. However, in contrast to patients with hemorrhagic stroke, therapy resumption was associated with a significantly lower rate of recurrent ICH [17].

Several guidelines with different levels of evidence have been published in the literature. As per the 2020 recommendations published by the European Society of Cardiology, restarting oral anticoagulants in Afib patients at high risk of ischemic stroke should be addressed in conjunction with a neurologist/stroke specialist after traumatic and spontaneous ICH. Non-vitamin K oral anticoagulants are preferred over vitamin K oral anticoagulants in eligible patients (Grade C). Anticoagulation after ICH should be postponed beyond the acute period for at least four weeks. In patients with Afib, who are at a very high risk of recurrent ICH, left atrial appendage closure may be considered (Grade B) [23].

In addition, the optimal time to resume anticoagulation therapy, as per the guidelines set forth by the European Heart Rhythm Association in 2021, is four to eight weeks after a comprehensive multidisciplinary evaluation. In cases where this does not occur, left atrial appendage closure is strongly advised [24].

According to the European Stroke Organisation (ESO)-Karolinska Stroke Update Conference, reinitiating oral anticoagulants may enhance prognosis in certain ICH patients without increasing the incidence of ICH recurrence (Grade C), in comparison to no oral anticoagulants. Non-vitamin K oral anticoagulants may be safer alternatives to vitamin K oral anticoagulants for ICH survivors with NVAfib (Grade C). The resumption of oral anticoagulants in NVAfib appears to be safe within the first four to eight weeks following the ICH index (Grade C) [25].

Dosing with direct oral anticoagulants following acute spontaneous ICH necessitates a meticulous assessment of the associated risks and benefits as per the 2018 CHEST guidelines. In addition, left atrial appendage closure is recommended for individuals who are specifically chosen and have a high risk of recurrent ICH, such as those who have concurrently diagnosed or suspected cerebral amyloid angiopathy [26].

Based on the ACC Expert Consensus, the temporal resumption of anticoagulation after ICH remains an area that lacks systematic investigation, and observational studies report considerable variation in timing (72 hours to 30 weeks), indicative of a lack of consensus.

On the other hand, guidelines advise against anticoagulation for at least four weeks in patients without mechanical heart valves; if necessary, monotherapy with aspirin may be resumed in the days following ICH. A large retrospective study found that resuming oral anticoagulants is associated with beneficial outcomes. On average, it took approximately one month after the occurrence of hemorrhage to resume oral anticoagulants. In patients without a high thrombotic risk, the writing committee recommends deferring the resumption of anticoagulation therapy for a minimum of four weeks [27].

Although our research utilized data from a tertiary medical center that treats a significant proportion of Afib cases in the Western region, it is essential to recognize its various limitations. The small sample size of this study and the short follow-up periods pose a challenge. Moreover, almost half of the patients died during hospitalization, further compromising follow-up findings. In addition, anticoagulation reversal strategies were not systematically assessed, and future studies should explore their impact on outcomes and the timing of anticoagulation resumption. Although this might have influenced the results, we hope that we can contribute to the literature by being one of the very few articles that highlight the practice in Saudi Arabia with ICH in Afib patients. Further multicenter studies with larger samples are warranted to provide more generalizable results.

## Conclusions

The decision to discontinue antithrombotic therapy in Afib patients with ICH has been an area of debate. It is necessary to strike a delicate equilibrium between the benefits and drawbacks of discontinuing and resuming antithrombotics. Our findings revealed a notable variation in the management of ICH in Afib patients, which suggests the absence of relied-upon guidelines. The outcomes can be poor, with high mortality rates.

The acute period is the most critical, and the prognosis appears to be favorable in patients who pass it. Utilizing a multi-specialist approach is essential for decision-making. The adaptation of clear guidelines to be followed in the management of such patients may improve overall outcomes and prognosis.
